# INHALE: the impact of using FilmArray Pneumonia Panel molecular diagnostics for hospital-acquired and ventilator-associated pneumonia on antimicrobial stewardship and patient outcomes in UK Critical Care—study protocol for a multicentre randomised controlled trial

**DOI:** 10.1186/s13063-021-05618-6

**Published:** 2021-10-07

**Authors:** Juliet High, Virve I. Enne, Julie A. Barber, David Brealey, David A. Turner, Robert Horne, Mark Peters, Zaneeta Dhesi, Adam P. Wagner, Alyssa M. Pandolfo, Sue Stirling, Charlotte Russell, Justin O’Grady, Ann Marie Swart, Vanya Gant, David M. Livermore, INHALE Study Group

**Affiliations:** 1 Norwich Clinical Trials Unit, University of East Anglia, Norwich, NR4 7TJ UK; 2 University College London, Gower Street, London, WC1E 6BT UK; 3 NIHR University College London Hospitals Biomedical Research Centre, University College London Hospitals NHS Foundation Trust, London, NW1 2PG UK; 4 University College London Great Ormond Street Institute of Child Health and Great Ormond Street Hospital NIHR Biomedical Research Centre, WC1N 3JH, London, WC1N 3JH UK; 5 Norwich Medical School, University of East Anglia, Norwich, NR4 7TJ UK; 6 Quadram Institute Bioscience, Norwich Research Park, Norwich, NR4 7UA UK; 7 University College London Hospitals NHS Foundation Trust, London, NW1 2PG UK

**Keywords:** Antibiotic stewardship, Randomised controlled trial, Hospital-acquired pneumonia, Ventilator-associated pneumonia

## Abstract

**Background:**

Hospital-acquired and ventilator-associated pneumonias (HAP and VAP) are common in critical care and can be life-threatening. Rapid microbiological diagnostics, linked to an algorithm to translate their results into antibiotic choices, could simultaneously improve patient outcomes and antimicrobial stewardship.

**Methods:**

The INHALE Randomised Controlled Trial is a multi-centre, parallel study exploring the potential of the BioFire FilmArray molecular diagnostic to guide antibiotic treatment of HAP/VAP in intensive care units (ICU); it identifies pathogens and key antibiotic resistance in around 90 min. The comparator is standard care whereby the patient receives empirical antibiotics until microbiological culture results become available, typically after 48–72 h. Adult and paediatric ICU patients are eligible if they are about to receive antibiotics for a suspected lower respiratory infection (including HAP/VAP) for the first time or a change in antibiotic because of a deteriorating clinical condition. Breathing spontaneously or intubated, they must have been hospitalised for 48 h or more. Patients are randomised 1:1 to receive either antibiotics guided by the FilmArray molecular diagnostic and its trial-based prescribing algorithm or standard care, meaning empirical antibiotics based on local policy, adapted subsequently based upon local microbiology culture results. Co-primary outcomes are (i) non-inferiority in clinical cure of pneumonia at 14 days post-randomisation and (ii) superiority in antimicrobial stewardship at 24 h post-randomisation (defined as % of patients on active and proportionate antibiotics). Secondary outcomes include further stewardship reviews; length of ICU stay; co-morbidity indicators, including septic shock, change in sequential organ failure assessment scores, and secondary pneumonias; ventilator-free days; adverse events over 21 days; all-cause mortality; and total antibiotic usage. Both cost-effectiveness of the molecular diagnostic-guided therapy and behavioural aspects determining antibiotic prescribing are being explored. A sample size of 552 will be required to detect clinically significant results with 90% power and 5% significance for the co-primary outcomes.

**Discussion:**

This trial will test whether the potential merits of rapid molecular diagnostics for pathogen and resistance detection in HAP/VAP are realised in patient outcomes and/or improved antibiotic stewardship.

**Trial registration:**

ISRCTN Registry ISRCTN16483855. Retrospectively registered on 15 July 2019

**Supplementary Information:**

The online version contains supplementary material available at 10.1186/s13063-021-05618-6.

## Background

Hospital-acquired and ventilator-associated pneumonias (HAP and VAP) are medically important owing to their frequency and high mortality and because they drive the use of broad-spectrum antibiotics. In the United Kingdom (UK), VAP occurs in 9–27% of ventilated patients, with an incidence of 5 cases/1000 ventilator days [1]. The cost is high: Edwards et al. [2] estimated around £19–20K per patient for severe pneumonia, even at 2008 prices.

Most ventilated patients are in intensive care, which has heavier antibiotic use and selection pressures than other hospital sites, with correspondingly greater resistance [3]. Crucially, in context, pulmonary infections account for approximately 50% of all ICU antibiotic use [1, 4], underscoring their contribution to these antibiotic pressures.

Timely treatment is crucial to outcome in HAP and VAP, with mortality increased if antibiotics are withheld or delayed [5]. Accordingly, antimicrobial chemotherapy is begun empirically, at clinical diagnosis, with the agents used being selected based on guidelines; local resistance rates; and patient risk factors for resistant bacteria (e.g. other recent antibiotics and duration of hospitalisation(s)). This approach reflects the fact that it takes 48–72 h to grow and test the bacteria causing the infection by a standard of care microbiology, delaying the opportunity to match the antibiotic to the pathogen present.

Treatment inadequacy, because the pathogen proves resistant to the empirical agent(s), is associated with increased mortality [6]. Consequently, fears of resistance-associated failure create pressure to empirically prescribe the broadest-spectrum antibiotics, including carbapenems [7] as recorded in a recent NHS longitudinal analysis [8]. This approach is argued to increase survival and to have health economic benefits (e.g. shorter time in ICU), including in NHS settings [2] but amounts to poor stewardship. Many patients with susceptible pathogens are given unnecessarily broad-spectrum antibiotics, and these exert pressure on the gut flora, favouring the overgrowth of drug-resistant bacteria, which constitute a reservoir of future opportunist pathogens.

Improved infection control has reduced the NHS’s burden of methicillin-resistant *Staphylococcus aureus* (MRSA) and *Clostridium difficile*, but resistance rates among Gram-negative bacteria are rising and, on an international basis, are doing so alarmingly, e.g. with an explosive increase of *K. pneumoniae* with KPC carbapenemases in Italy and Greece and Enterobacterales with NDM carbapenemases in the Indian subcontinent [9]. Most carbapenemase producers are susceptible in vitro only to a few antibiotics, e.g. colistin and tigecycline, that have significant toxicity and efficacy limitations.

The conventional approach to overcoming resistance has been the development of new antibiotics, but the flow of these has slowed, reflecting the difficulty of discovering, developing and licensing them and the low-return on investment. Although new antibiotics, covering some multi-resistant Gram-negative pathogens, are beginning to reach the market, none wholly overcomes the existing resistance and all are vulnerable to new resistance. Restrictive stewardship conserves existing antibiotics, but at a risk of denying effective treatment to seriously ill patients. As recognised in the Chief Medical Officer’s (CMO) Report [10], it is vital to find alternative, evidence-based models to guide the use of both new and established antibiotics. These logically include rapid diagnostics, and in his 2016 report commissioned by the UK government, O’Neill [11] recommended that, by 2020, antibiotics should only be prescribed if informed by data and testing technology.

In principle, and even without rapid diagnostics, the broad-spectrum empirical therapy initiated when HAP or VAP is clinically diagnosed should be de-escalated to narrower-spectrum therapy once the pathogen is identified and its resistances determined by the microbiology laboratory, a process that typically takes 48–72 h. This strategy, sometimes dubbed ‘start smart, then focus’, has developed over 70 years, and its timescales depend upon the speed of bacterial growth and testing in the microbiology laboratory. Although sound in principle, this approach has three major limitations.

First, as already noted, it leads to over-treatment of the many patients with susceptible pathogens, unnecessarily increasing the pressure on the gut flora.

Secondly, many patients with clinically diagnosed infections have no pathogen grown. This proportion is as high as 70% in pneumonia [12]. Failure to grow a pathogen may reflect suppression of growth by antibiotic(s) already given to the patient, inappropriate culture technique or a purely viral aetiology. Since their pathogens remain undefined, and with the threat of increased mortality due to under-treatment, these patients often spend prolonged periods on broad-spectrum empirical agents, including empirical carbapenems. This raises the contingent risk of side effects and selection of a resistant gut flora. Even when microbiology results become available, indicating susceptible pathogens, clinicians frequently fail to de-escalate broad-spectrum therapy. This can be unintentional, reflecting other aspects of the critically ill patient’s treatment being prioritised. It can also be intentional, especially when the patient is doing well clinically, with doctors reluctant to change a ‘winning’ therapy lest the patient deteriorates again [13]. These issues are exacerbated by long waits for microbiology results.

Thirdly, even with broad-spectrum agents, empirical therapy is likely to prove inadequate in patients with unusually resistant pathogens, whose mortality risk is thereby increased in severe infection [14]. Peralta et al. [15] found that the risk of empirical treatment proving to be inappropriate rose from 3% for patients with pathogens lacking resistance to 35% for those resistant to 3 or more antibiotic classes, with a commensurate increase in deaths. In normal (i.e. non-pandemic) circumstances, such under-treatment is most likely in centres providing tertiary care and serving mobile populations with extensive travel to countries with higher resistance rates (e.g. many central London teaching hospitals) and in those private hospitals that treat patients from regions where resistance is highly prevalent (e.g. in the Middle East). In the longer term, the risk of under-treatment is likely to increase and become more widespread, especially if, as seems likely, the accumulation of resistance continues to outstrip antibiotic development, particularly against Gram-negative opportunist pathogens.

Molecular microbiological diagnostics offer a potential route to overcoming these limitations by identifying the pathogens and their resistances in hours instead of days, allowing immediate targeted therapy or, at least, much earlier therapeutic refinement. Several automated, polymerase chain reaction (PCR)-based pathogen and resistance detection platforms are now available for microbiological evaluation of HAP and VAP patients, but no data exist on whether these offer advantages in respect of clinical outcomes, or if they are cost-effective.

The INHALE Randomised Controlled Trial (RCT) detailed here is Work Package 3 (WP3) of the NIHR-funded INHALE Research Programme. In WPs 1 and 2, three rapid diagnostic systems—the PCR-based BioFire FilmArray, Curetis Unyvero platforms and rapid sequencing with the Oxford Nanopore Technologies MinION—were evaluated on respiratory specimens from ICU patients ([16, 17]. Based on the results, the FilmArray Pneumonia Panel (the ‘FilmArray test’) was selected as the best performing test to carry forward into the present RCT. Prior evaluation of the FilmArray test was extremely limited at the start of this work, with no UK study. In addition, and of particular importance, there was (and is) no evidence base to direct how and where to deploy such a platform within clinical pathways for maximum efficiency and value for money. Whether, how and why physicians might welcome such tests—which deliver swifter results at the expense of a more restricted range of bacterial pathogens and (especially) resistances detected—were unknown. Addressing these behavioural factors of ‘acceptability’ is essential for effective implementation, assuming the present trial finds the FilmArray test advantageous.

Accordingly, the INHALE RCT is a two-armed study with two co-primary outcomes: non-inferiority in clinical cure of pneumonia at 14 days post-randomisation and superiority in anti-microbial stewardship at 24 h post-randomisation. In the intervention arm, therapy for HAP/VAP will be guided by the FilmArray test, undertaken within ICUs rather than in a laboratory; the results are linked to an algorithm to translate its outputs into treatment guidance. In the control arm, patients will receive standard empirical antibiotics, according to the local prescribing policy. Both arms allow further refinement of therapy once conventional culture-based results become available.

Behavioural analysis will (i) compare the clinicians’ decision-making with and without the FilmArray test, (ii) explore clinicians’ barriers to adopting molecular diagnostics and (iii) provide evidence-based recommendations for how to overcome these. An economic analysis will review the cost-effectiveness implications.

## Objectives

The overall trial aim is to show that clinical and safety outcomes for patients whose treatment is guided by the FilmArray test molecular diagnostic are non-inferior compared to standard care, but that altered prescribing leads to improved antimicrobial stewardship.

### Primary objectives

The co-primary objectives are as follows:

1. To determine whether there is non-inferiority in clinical cure of pneumonia at 14 days post-randomisation between patients treated according to the FilmArray test’s molecular results plus trial-based prescribing algorithm versus those treated with standard care.
2. To determine whether there is an improvement in antimicrobial stewardship at 24 h post-randomisation for participants treated according to the FilmArray test versus those treated with standard care. In context, antimicrobial stewardship is defined as the receipt of active and proportionate treatment.

Since these are co-primary objectives, the study will be declared to have met its primary objectives only if the FilmArray test is found to be both non-inferior to standard care in terms of clinical cure and also provides improvements in antimicrobial stewardship.

### Secondary objectives

FilmArray and standard care arms additionally will be compared to determine whether:

1. There is a difference in the number of participants receiving an appropriate antibiotic at 24 and 72 h post-randomisation
2. There is a difference between the two groups in total antibiotic use over the 21-day study period
3. The FilmArray test plus algorithm is more cost-effective than the standard care at 21 days post-randomisation
4. There are any differences in antibiotic-associated adverse events (e.g. *C. difficile* infection) between the two groups within 21 days of randomisation
5. There are changes in organ dysfunction scores between the two groups at day 7 post-randomisation
6. ICU/critical care unit (CCU) length of stay, septic shock rates, or mortality rates are decreased (or increased) by the intervention compared to the standard care
7. There is an increase in ventilator-free days for ventilated participants in the intervention group
8. There are differences between the groups in the number of participants contracting secondary infections

## Methods/design

### Design

INHALE WP3 is a multi-centre, open-labelled, parallel, randomised controlled trial exploring the potential impact of the FilmArray rapid molecular diagnostics coupled with a prescribing algorithm, aiming for non-inferiority in clinical cure of pneumonia and superiority with regard to antimicrobial stewardship, compared with standard care. Additional file 1: Figure S1 illustrates the design. Participants are randomised in a 1:1 ratio centrally, via a Research Electronic Data Capture (REDCap) [18, 19] web-based randomisation system, to receive either the intervention plus standard care or standard care alone. Randomisation is stratified by site using permuted block allocation of randomly varying block lengths; concealment of allocation is guaranteed by using the web-based system. All participants remain in the trial for up to 28 days for data collection. Once a participant is randomised, the trial is open-label, meaning clinicians and all study team members will know which group a participant is in. The participant will also be informed upon request. Outcome assessors of the superiority co-primary outcome will remain blinded to the treatment group, and there are no circumstances under which allocation will be revealed to them.

### Intervention

The trial intervention under test is treatment guided by the BioFire FilmArray Molecular Diagnostic Machine Pneumonia Panel (the ‘FilmArray test’), a PCR-based system for identifying pathogens causing lower respiratory tract infections (LRTI) and their critical antibiotic resistance genes, coupled with a trial-based prescribing algorithm adapted to accommodate appropriate site-specific requirements. The FilmArray tests are performed in the ICU; they have a set-up time of approximately 2 min and a run time of 1 h 15 min. The control arm is standard care, which consists of empirical antibiotics, based on local policy.

Both arms have standard microbiology culture and susceptibility testing performed, according to local laboratory procedures, with results typically available after 48–72 h. These results allow further refinement of therapy in both arms.

To allow both FilmArray and conventional investigation, all randomised participants require an appropriate respiratory specimen (sputum, endotracheal aspirate, bronchoalveolar lavage (BAL) or non-bronchoscopic lavage (NBL)) for microbiological investigation, collected within ±12 h of the decision to change or initiate an antibiotic for suspected LRTI. The specimen is collected into two separate containers to create two identical samples, designated sample 1 and sample 2. For all participants, sample 1 follows the usual care pathway for respiratory specimens, and microbiology results are reported when available, as standard. Sample 2 is labelled with the INHALE trial identifiers and follows one of two routes dependent upon the treatment group.

For participants in the intervention arm, sample 2 is tested with the FilmArray Pneumonia Panel in the ICU as soon as possible (in all cases within 24 h). The results are reviewed, as soon as they are available, by the treating clinician, whose antibiotic prescription is directed by the trial-specific algorithm and local prescribing guidelines. It is anticipated that the great majority of intervention participants will receive either only one dose of empirical treatment or perhaps none before the FilmArray test result allows treatment to be targeted. The participant will continue receiving normal clinical care. For participants in the control arm, sample 2 is frozen within 24 h, and the FilmArray test is run by either of the two central laboratories located at the University College London and University of East Anglia, without the result being made known to the treating clinicians. Where consent is given, the spare sample may be retained for future use in ancillary studies.

Future specimens from the same intervention group participant may be tested using the FilmArray test provided the following conditions are met: (i) the participant has not withdrawn from the trial; (ii) the specimen is taken as part of the routine care, and a matching specimen (sample 1) is sent to the microbiology laboratory; (iii) testing is no less than 72 h after the previous specimen and would have been taken regardless of the trial; (iv) there is enough specimen for the machine testing; and (v) the participant was randomised to the trial ≤ 21 days previously.

As the intervention happens immediately after randomisation, no special strategies to improve adherence are specified in the trial. All standard care options are available to clinicians at all times. Barriers to algorithm adherence are explored and analysed as part of the behavioural study. Therefore, there are no special criteria for discontinuing or modifying the allocated interventions.

### Setting

The trial is being run across ICUs at multiple (provisionally 12) hospitals with different resistance prevalence rates, environments and case mixes. They include London teaching hospitals, regional teaching hospitals, district hospitals, specialist children’s hospitals and a private hospital with an international patient mix. They have ICUs handling various proportions of medical, surgical and trauma cases. ICU sites are responsible for identifying, recruiting and consenting participants. Participants will remain under the care of their usual health services throughout their time on the trial (see ISRCTN registration for a current list of participating sites).

### Treatment algorithm

We drafted a master prescribing algorithm based on the pathogens sought by the FilmArray Panel and current UK resistance prevalence data as represented in the British Society for Antimicrobial Chemotherapy surveillance of respiratory pathogens [20]. Site microbiologists, ICU pharmacists and ICU clinicians were consulted; local adaptations were allowed, particularly in paediatric practice, where fewer relevant antibiotics are licensed. Where possible the algorithm [see Additional File 2] vocates narrow-spectrum agents: e.g., temocillin versus Enterobacterales and flucloxacillin versus MRSA. Alternatives are offered for patients with mild or severe (anaphylaxis risk) β-lactam allergy, with the former allowing non-penicillin β-lactams and the latter eschewing β-lactams entirely. Where combinations of organisms are detected, these are sorted hierarchically, with a ‘base’ agent advised to cover the most inherently resistant species and additions to cover the further organisms or resistances. Once local algorithm variations had been agreed upon with senior site ICU physicians and microbiologists, we provided training to a wider group of site ICU staff, including physicians and research nurses. This was done face-to-face, using example FilmArray reports, and with group teaching on how to interpret the algorithm and which antibiotics to use.

### Eligibility criteria

The following are the inclusion criteria:

1. About to receive an antimicrobial to treat a suspected LRTI—being either (i) first treatment of a newly suspected HAP/VAP or (ii) a change in previous antimicrobials for an LRTI owing to the deteriorating clinical condition
2. In-patient in a participating ICU/CCU
3. Breathing spontaneously or are intubated for any reason
4. Hospitalised for > 48 h
5. Able to provide sufficient volume of airway specimen obtained for routine testing plus 200 μL for the FilmArray test (samples 1 and 2 above)

The following are the exclusion criteria:

1. Have previously taken part in this trial.
2. Concurrently participating in the active phase (defined as within 30 days of the primary end point) of an interventional trial not agreed as acceptable for co-enrolment by the local PIs of both trials. Participants will be permitted to co-enrol in studies that do not involve an intervention (e.g. observational studies).
3. Moribund and/or not expected to live for more than 48 h.
4. Have an existing directive to withhold life-sustaining treatment, in relation to antibiotic use.
5. Prisoners or young offenders currently in the custody of the Prison Service or supervised by the Probation Service.

Participants with COVID-19 can be recruited to the trial, providing all other criteria are met, critically including antibiotic initiation or change predicated on concern about secondary bacterial infection. This is reflective of the current ICU population and aims to make trial results as generalizable as possible.

### Recruitment and consent

Recruitment of participants is from pre-approved ICUs in England. Participants cannot self-refer; rather, they are identified as potentially eligible by clinicians in the ICU.

Pneumonia and other LRTIs in the ICU are life-threatening, and the decision to administer antibiotics is taken very quickly; this decision must not be delayed because of the trial, precluding any attempt to collect fully informed consent prior to inclusion and treatment or to elicit consent from distressed relatives. Consent is therefore not taken before randomisation and initial delivery of the intervention, but instead follows a retrospective process. This is recognised in the European Law [21], and this decision has been made in consultation with the trial Patient and Public Involvement (PPI) panel.

Specifically, consent or (more often) assent is taken at the earliest, or most appropriate, opportunity after the initial treatment on day 1 and preferably within the next 48 h. Participants or their consultees (in England and Wales; an unpaid person with an interest in the welfare of the potential participant or a healthcare professional who is independent of the project) are given as long as needed to make an informed decision and the participant remains in the trial whilst a decision is sought and as long as there is no objection. The consultee may be a personal consultee or an independent professional consultee.

In most cases, it is anticipated that a consultee will be approached to give assent due to the expected incapacity of most ICU/CCU patients to give consent. To enable such patients’ participation in clinical research, the Mental Capacity Act (2005) [22] allows a consultee to grant/withhold permission until the participant recovers capacity. Should the participant recover capacity, they will be approached about the trial directly, and in this circumstance, their consent/refusal overrides the consultee agreement.

For children (aged 15 years or under), the parents or guardians of a child are approached to give consent for their child to participate. Participation is refused in the event that a child is distressed by participation or does not assent when they recover capacity.

### Data collection

Participants have data collected from their routine care records, for assessments as shown in Additional file 3: Table S1.

Data collection continues from day 1 to 21 days after randomisation or until death if earlier. Daily assessments stop at day 14, or earlier if the participant is discharged from the ICU/CCU or dies before day 14. For participants discharged home prior to 21 days post-randomisation, a brief telephone interview is conducted between days 20 and 24, providing consent is given. The condition of participants still in hospital at 21 days is assessed from their notes. A medical record check is carried out for the participant’s final date of discharge, or death noted up to day 28.

Study data are collected and managed using REDCap [18, 19] electronic data capture tools hosted at the Norwich Clinical Trials Unit (NCTU). Research Electronic Data Capture (REDCap) is a secure, web-based software platform designed to support data capture for research studies, providing audit trails for tracking data manipulation and export procedures and automated export procedures for seamless data downloads to common statistical packages.

### Adverse event reporting and monitoring

Adverse events (AEs) and serious adverse events (SAEs) that are secondary outcomes in the trial do not need to be reported in an expedited manner but are recorded in the database. Machine and laboratory errors or issues producing misleading or wrong results, leading to ineffective therapy with potential or actual serious consequences, are to be reported immediately as SAEs. Such errors may occur as a result of technical problems or by limitations of the design (e.g. organisms not present on the panel or specific laboratory reporting practices). In addition, investigators may report other relevant events.

An independent Data Monitoring Committee (DMC) has oversight of all trial safety data and can make recommendations as required. No interim analyses are planned.

Adherence to the protocol is monitored throughout, as are data quality and completeness.

### Outcomes

The trial has co-primary outcomes of the following:

1. Non-inferiority in clinical cure of pneumonia at 14 days post-randomisation. Cure of pneumonia is defined as follows: absence of (i) death where pneumonia was considered causative or contributory; (ii) septic shock, except when associated with a documented non-respiratory origin of infection; (iii) relapse of pneumonia (relapse is defined as an infectious pulmonary event, associated with clinical and radiological signs of HAP or VAP, or a worsening of 2 points of the baseline multiple organ dysfunction score (SOFA or PELOD-2)); or (iv) other evidence that the original pneumonia is not cured.
2. Superiority in antimicrobial stewardship at 24 h post-randomisation is defined as participants on active and proportionate antimicrobial therapy within 24 h of clinical diagnosis, where active therapy is defined as receiving an antimicrobial active against the organism(s) in vitro and proportionate as active and not excessively broad spectrum for the pathogen(s) identified.

The secondary outcomes comprise the following:

(i) ICU/CCU length of stay, calculated as the time from randomisation to discharge from the ICU/CCU
(ii) Number of ventilator-free days within 21 days post-randomisation
(iii) Mortality, calculated as deaths from any cause within 28 days of randomisation
(iv) Incidence of septic shock within 21 days of randomisation
(v) Change in SOFA (ΔSOFA) score from randomisation to 7 days post-randomisation for adults
(vi) Change in PELOD-2 (ΔPELOD-2) score from randomisation to 7 days post-randomisation for children
(vii) Change in pSOFA (ΔpSOFA) score from randomisation to 7 days post-randomisation for children
(viii) Proportion of participants on antibiotics active/inactive against the pathogen(s) found at 24 and 72 h from randomisation
(ix) Proportion of participants on proportionate/disproportionate antibiotics, in relation to pathogen(s) found at 72 h from randomisation
(x) Proportion of participants on narrow-spectrum antimicrobials at 24 and 72 h from randomisation
(xi) Proportion of participants with specific adverse events associated with antibiotics within 21 days from randomisation
(xii) Proportion of participants contracting a secondary pneumonia within 21 days from randomisation
(xiii) Total per patient antibiotic usage in defined daily doses (DDDs) by 21 days post-randomisation (all conditions)

### Sample size

The trial aims to recruit 552 patients over a 24-month recruitment period. Calculation of the required sample size is based on the co-primary outcomes of clinical cure at 14 days and antimicrobial stewardship at 24 h. The trial will investigate non-inferiority in terms of clinical cure and superiority for antimicrobial stewardship. Calculations aim to achieve 90% power and 5% significance for the co-primary analyses and allow for up to 5% attrition. Data for the first 100 patients from work package 2 provide estimates for the sample size calculation. For the non-inferiority outcome, a clinical cure rate of 55% is assumed in both trial arms, and the non-inferiority limit is defined to be 13% [23–27]. Under standard care, it was estimated that 53% of patients received antibiotics that were both appropriate and proportionate within 24 h of clinical diagnosis, and it is clinically important to improve this by at least 20% (to 73%). The planned sample size of 552 patients will provide 91% power for the non-inferiority outcome analysis and 99% power for the superiority outcome, resulting in 90% for the co-primary analysis (0.91 × 0.99 = 0.9), under the conservative assumption of no correlation between the outcomes [28]. The sample size is not inflated for non-compliance, as none is expected.

The estimates used for both cure rate and antimicrobial stewardship are consistent with those reported in published studies [24–30].

The trial originally aimed to recruit 466 participants, but this was updated to 552 to reflect an expected lower estimated cure rate for pneumonia among COVID-19 patients (postulated at the time to be between 20 and 40% vs. an original estimate, pre-COVID, of 70%). Assuming a maximum of 30% of the final sample might be COVID-19 patients, the minimum expected overall cure rate is 55%, as used in the new sample size calculation. Estimates were based on the data already collected and decided in consultation with the principal investigators.

### Analysis

The co-primary outcomes will be summarised as the proportion of participants where:

- Clinical cure was achieved by 14 days after randomisation;
- Active and proportionate antimicrobial therapy has been initiated within 24 h of randomisation.

For both outcomes, the effect of the intervention will be described using a difference in proportions, and an odds ratio, each calculated with a 95% confidence interval. For the non-inferiority analysis of clinical cure, confidence intervals will be one-sided. Estimates will be obtained from regression models that allow for study site. Similar approaches will be used for binary secondary outcomes. For continuous secondary outcomes, the groups will be compared using standard regression models (where normality assumptions are satisfied) to obtain the differences in the means allowing for site and adjusting for baseline values where these are available.

In all superiority analyses, participants will be analysed on an intention-to-treat basis, regardless of clinicians’ decisions in using the FilmArray test results and the associated algorithm, or in respect of the antibiotics prescribed. A Microbiology Committee, comprising three independent members and one member of the study team, has been set up to review the prescribing decisions. The committee assesses whether prescribing is active and proportionate at 24 h and 72 h post-randomisation for each patient and is blinded to the study arm. Per-protocol analysis will provide the primary results for the non-inferiority outcome. The committee Terms of Reference are shown [see Additional File 4].

Data analysts will not be blinded. A detailed statistical analysis plan will be finalised before analysis and will include details of any relevant sub-group or adjusted analyses. It will be made available, upon request, from the Trial Management Group.

### Health economics evaluation

To determine whether the trial outcomes justify costs, a cost-effectiveness study will be conducted from a hospital perspective. The outcome measure used will be the same as the main study co-primary outcome. If the two arms of the study are found to be equivalent in terms of clinical cure of pneumonia at 14 days post-randomisation, a cost-effectiveness analysis will be undertaken to evaluate the incremental cost per additional patient receiving active and proportionate antibiotics within 24 h of clinical decision to prescribe antibiotics for HAP/VAP. If equivalence in clinical cure is not established but there is an improvement in antibiotic stewardship, interpretation will be more difficult—a decision-tree model will be used to explore the implications.

The EuroQol EQ-5D-5L, a generic health-related quality-of-life measure [31], will be sought from patients at 21 days to inform the economic evaluation and economic model, but will not be suitable for estimating quality-adjusted life-years (QALYs) for all trial participants. This is because only the most-recovered subset of participants will be well enough to complete the EQ-5D-5L at 21 days, meaning that results will not be representative of the whole INHALE sample.

Data collection for resource use will be limited to the patients’ hospital stay, meaning that an ‘NHS and personal social services perspective’, as recommended by NICE [32], will not be possible. Costs will be estimated solely from the perspective of the hospital.

Estimates of costs and effects will be used to calculate the incremental cost-effectiveness ratios (ICERs), evaluating the incremental cost per additional patient receiving active and proportionate antibiotics within 24 h of the decision to prescribe antibiotics for HAP/VAP. Where appropriate, regression-based methods will be used to allow for differences in baseline characteristics. Uncertainty in data will be accommodated by the use of cost-effectiveness acceptability curves (CEACs), which estimate the probability that the intervention is cost-effective at different monetary valuations of the outcome measure. As costs will be estimated from routinely collected hospital data, we would not expect high rates of missing data. This approach constitutes a ‘within-trial’ economic analysis, conducted during the clinical trial, and will constitute the primary economic analysis.

As the study population will be highly heterogeneous in respect of underlying illness or trauma precipitating ICU admission, economic evaluation may not be straightforward. Additionally, the trial will take place within a short timeframe, so long-run outcomes will not be available. Rather, to explore the effect of assumptions and potential variability, a decision-tree model will be further developed across the entire INHALE programme. Data from the literature will be used to estimate the potential long-run health-related quality of life (HRQoL) effects of HAP/VAP, as well as estimating a cost/QALY approach. Assumptions as to the health benefits of different pathways and outcomes will allow an exploration of potential longer-term effects (e.g. potential effects on antibiotic use, resistance and related health outcomes). Literature data [33] and clinical opinion will be incorporated where necessary. This decision-tree model will be used to explore a range of ‘what if’ scenarios, evaluating different consequences of results from the trial. Assumptions about the consequences of care provided and of improved antimicrobial stewardship will be formulated. This approach is speculative but will potentially inform decision-makers as to the potential consequences of rapid microbiological diagnostics. The results of this model will constitute a secondary analysis. Details of the proposed methods for the within-trial analysis and the economic model will be pre-specified in a health economics analysis plan (HEAP).

### Behavioural evaluation

Examining how clinicians make antibiotic decisions in both arms of the trial is crucial to (i) identifying the behavioural factors influencing antibiotic prescribing (whether appropriate or not); (ii) quantifying the degree to which the FilmArray test and algorithm influences doctors’ antibiotic decisions, as to assume 100% compliance with its recommendations is neither appropriate nor realistic; and (iii) providing a context for RCT findings. Prescriber behaviour will be a key determinant of the trial outcomes. Rapid molecular diagnostics can only improve antibiotic stewardship if they are appropriately applied. Understanding the drivers of, and barriers to, adoption of rapid molecular-diagnostic-assisted prescribing (e.g. prescriber trust in FilmArray results) will help identify the reasons behind trial success/failure.

Data will be collected using a 1-min questionnaire, developed for INHALE and completed after a specific antibiotic decision. This methodology allows for a range of decisions (e.g. from sites across the UK, out-of-hours decisions) to be consistently captured with minimal disruption to clinical duties. In the intervention arm, the questionnaire explores the decision made after receiving the FilmArray test results. In the control arm, it relates to the empirical antibiotic decision made directly before randomisation. The clinician who made the ultimate decision will be asked to complete the survey: intensivists and specialists working in the ICU will be eligible to participate. To minimise recall bias [34], the clinician will complete the survey as soon as possible after this decision, but no later than 24 h afterwards. The questionnaire asks the clinician to indicate factors influencing their decision-making and rate their agreement with statements about their patient as well as the role of institutional guidelines, the FilmArray test and the algorithm. Survey design was a collaboration between behavioural scientists, intensivists and microbiologists and was informed by a literature review, four focus groups and 35 interviews with ICU prescribers.

We will also conduct semi-structured interviews with clinicians to further explore their perceptions about using molecular diagnostics in practice. Participants will be intensivists and microbiologists who have made antibiotic decisions with the FilmArray during the RCT.

Clinicians will be encouraged to participate via the principal investigator at each site, with interview transcripts anonymised. ICUs are time-pressured environments, and participation rates will be monitored and changes made if the design proves difficult, but the team are experienced, and the proposal is based on feasibility work from earlier work packages. Thematic analysis [35] will be used to identify clinicians’ perceptual and practical barriers and enablers to adopting the FilmArray test and the algorithm. Our findings will aid the development of evidence-based behavioural strategies encouraging molecular diagnostic implementation.

### Oversight and monitoring

Oversight of the trial is managed by committees, each with a specific composition and remit, as described in their Terms of Reference, and summarised as follows.

#### Trial Management Group (TMG)

Together with the Trial Steering Committee (TSC), this has the overall oversight of the trial. Chaired by the chief investigator, it includes (i) clinicians from a broad range of trial sites; (ii) statistician, programme manager, behavioural study and health economics lead; (iii) members of the Norwich CTU-based trial team; (iv) sponsor representation; and (v) PPI representatives. All trial oversight groups report back to the TMG which meets at least every 6 months, though typically every 3 months during recruitment.

#### Trial Steering Committee (TSC)

The Programme Steering Committee (PSC) has agreed to also take up the TSC role, and so is kept appraised on overall trial progress. Through its independent chair, the TSC provides advice on all aspects of the trial to the TMG, sponsor, NIHR as funder and Norwich CTU. The DMC provides an independent update on data monitoring and safety directly to the TSC chair for consideration at each meeting. The TSC is composed of five independent members, including the chair, a PPI representative and three non-independent members (the chief investigator, trial statistician and NCTU director). The TSC meets at least annually, after DMC meetings, but more frequently during recruitment.

#### Patient and Public Involvement (PPI) group

The INHALE Public and Patient Involvement Group (comprising seven members) meets quarterly to discuss the programme progress and output; this also includes patient-facing documentation and reviewing all aspects relating to the management of the INHALE trial. In addition, two PPI panel members are part of the TMG, and there is PPI representation on the TSC.

## Discussion

ICUs have greater per patient antibiotic use, and so exert more selection pressures for resistance, than elsewhere in hospitals, with some (albeit mixed) evidence of correspondingly higher resistance rates [3]. Sub-optimal treatment leads to poorer outcomes for patients, including side effects from over-treatment and increased mortality from under-treatment of severe and resistant infections. The cost to society is also high, both in human terms and in respect of the cost of hospital stays.

Current microbiology techniques to identify pathogens and their resistance are slow and outdated and do not always grow a pathogen despite clinical evidence of infection. There are few new antibiotics reaching the market, and recently licensed ones do not wholly overcome resistance; moreover, they are vulnerable to new modes of resistance. The UK government’s ‘Contained and Controlled, 20-year vision for antimicrobial resistance’ [36] confirms the vital need to find alternative, evidence-based models for antibiotic use.

Molecular diagnostics, such as the FilmArray Pneumonia Panel, offer the potential to achieve this goal by rapidly and simultaneously identifying organisms and critical resistance genes directly from the clinical specimen, without culture. The partner algorithm being trialled together with the FilmArray Panel offers the potential to identify a proportionate antibiotic choice for each identified pathogen and resistance genotype detected—if clinicians are willing to adopt this approach in challenging and severely ill ICU patients. If successful, this work could be extended to other clinical settings, though the cost advantage, which will be analysed, is likely to be greatest in ICUs.

In addition, this trial will, to our knowledge, be the first to explore the human factors that interpose between a rapid microbiological result and the clinicians’ contingent actions that follow (or not). It has been designed to capture both the extent of clinicians’ compliance with the result-driven recommendations, and the reasons underlying such compliance, or lack of it.

Another novel element of the trial relates to linking a microbiology result to a suggested antibiotic prescription, as suggested by the algorithm, without human intervention in the decision pathway. To our knowledge, this is the first instance of advice being developed to translate the outputs of a complex multiplex microbiological test into prescribing guidance. Whilst it was not possible to impose a single algorithm at all hospitals, core principles are successfully retained. We believe this element of the trial is unique. Not only will the trial test the utility of the test and algorithm but, in addition, the behavioural sub-study will reveal new information on doctors’ attitudes to conventional and machine-driven results and recommendations.

## Trial status

Recruitment began on 5 July 2019; it was paused between March and July 2020 due to the COVID-19 pandemic and resumed in August 2020. The pause for COVID-19 means that recruitment will be extended beyond the original end date of 31 March 2021, stopping once the sample size is reached. The current protocol version is 3.0, dated 9 February 2021. An amendment was required to confirm that COVID-19-positive patients can now be included routinely in the trial, and this changed the sample size; the protocol has been amended previously to add sub-studies (to be reported separately).

## Supplementary Information

**Additional file 1: Figure S1**. Flow Diagram of Trial Design.

**Additional file 2:.** INHALE WP3 Antibiotic Prescribing Guidance v2.0 8May2019 MASTER; Algorithm.

**Additional file 3: Table S1**. Timeline for Trial Actions. *Later on day 1 and as soon as possible after decision to test for pneumonia. Further specimens from the same participant may be tested on the machine, within the 21-day trial period, only if clinically indicated and a sample has also been sent routinely to the microbiology laboratory. ^1^Every day until 14 days after randomisation or until clinical cure of pneumonia, whichever is first. Assessments only required on these days if in ICU/CCU and not cured of pneumonia. ^2^Which assessment is used depends on whether participant is a child or an adult. Clinical teams record these routinely and will know which should be used. ^3^Data collected may pre- and post-date the trial period, but will be collected from clinical records by hospital staff. ^4^Window for “day 21” phone call to occur is from days 20-24 post randomisation, discharged participants only. ^5^Record if relevant until 21 days after randomisation (even if pneumonia is cured sooner).

**Additional file 4:.** INHALE Micro Committee Terms of Reference v2.0 27JUN2021.

## Data Availability

The final trial dataset and associated trial documents such as master copies of patient information and consent forms will be made available, upon reasonable request to the Trial Management Group, once analysis and primary publication are complete.

## Abbreviations

HAP: Hospital-acquired pneumonia
VAP: Ventilator-associated pneumonia
UK: United Kingdom
RCT: Randomised controlled trial
ICU: Intensive care unit
LRTI: Lower respiratory tract infection
ISRCTN: International standard randomised controlled trials number
NHS: National health service
WP: Work package
CMO: Chief medical officer
PCR: Polymerase chain reaction
CCU: Critical care unit
NCTU: Norwich clinical trials unit
PI: Principal investigator
PPI: Patient and public involvement
DDD: Defined daily dose
SAE: Serious adverse event
DMC: Data monitoring committee
SOFA: Sequential organ failure assessment
NICE: National institute for health and care excellence
ITT: Intention-to-treat
TMG: Trial management group
PSC: Programme steering committee
TSC: Trial steering committee
