# Supplementary material for: INHALE: the impact of using FilmArray Pneumonia Panel molecular diagnostics for hospital-acquired and ventilator-associated pneumonia on antimicrobial stewardship and patient outcomes in UK Critical Care—study protocol for a multicentre randomised controlled trial
Source: Trials. 2021 Oct 7;22:680. doi: 10.1186/s13063-021-05618-6 (PMC8496625; doi:10.1186/s13063-021-05618-6)
Supplement: Supplementary file 1 — Additional file 1: Figure S1. Flow Diagram of Trial Design. [file 13063_2021_5618_MOESM1_ESM.docx]

Figure 1

Patient about to receive a new antimicrobial to treat a suspected LRTI – including suspected HAP/VAP, for the first time, or a change in antimicrobial for LRTI because of deteriorating clinical condition.

ICU/CCU patient in approved INHALE site and meets eligibility criteria

Day 1

Specimen taken for LRTI testing according to usual standard of care and either split immediately to create 2 samples or collected directly into 2 containers* (Sample 1, Sample 2).

Initial empirical antibiotics given according to local procedures (can be prior but must be within 12 hours of specimen collection)

Sample taken for LRTI testing according to usual care and either split immediately to create 2 samples or collected directly into 2 containers* (Sample 1, Sample 2).

233 participants

233 participants

Randomisation

Test **Sample 2** on FilmArray Test according to INHALE WP3 Lab Manual

**Intervention**

**Control**

Freeze **Sample 2** according to INHALE WP3 Lab Manual

Obtain data for all participants for their duration in the trial study

Send **Sample 1** to microbiology lab for culture and susceptibility testing (usual care)

Send **Sample 1** to microbiology lab for culture and susceptibility testing (usual care)

Review result and use algorithm to modify treatment if appropriate. Continue to monitor symptoms whilst awaiting microbiology result

S

Microbiology result received and reviewed, modify treatment according to usual practice and susceptibilities

Microbiology result received and reviewed, modify treatment according to usual practice and susceptibilities

Continue to collect data for 21 days (up to 14 days for daily assessments) or until clinical recovery (if earlier). On day 21, complete final assessment and if pneumonia is cured within 21 days, collect EQ-5D-5L on day 21 if appropriate.

Day 28

Day 28 –mortality assessment from patient notes

Ship samples when requested, to INHALE team

*samples must be same sample type and collected at the same time point
