# Supplementary material for: INHALE: the impact of using FilmArray Pneumonia Panel molecular diagnostics for hospital-acquired and ventilator-associated pneumonia on antimicrobial stewardship and patient outcomes in UK Critical Care—study protocol for a multicentre randomised controlled trial
Source: Trials. 2021 Oct 7;22:680. doi: 10.1186/s13063-021-05618-6 (PMC8496625; doi:10.1186/s13063-021-05618-6)
Supplement: Supplementary file 2 — Additional file 2:. INHALE WP3 Antibiotic Prescribing Guidance v2.0 8May2019 MASTER; Algorithm. [file 13063_2021_5618_MOESM2_ESM.pdf]

INHALE WP3: Antibiotic Prescribing Guidance for use with FilmArray Result

CONSIDERATIONS

1. **Adjust dosages for renal function as per Manufacturer's SPC**
2. **For patients already on antimicrobial treatment for infections necessitating specific regimens e.g. infective endocarditis/ meningitis - please discuss with Microbiology how best to adapt their treatment for organism(s) found by FilmArray.**
3. **Pregnant and paediatric patients: Please note general recommendations regarding use of fluoroquinolones, tetracyclines, colistin, temocillin and ceftazidime-avibactam. See https://www.medicines.org.uk/emc/ for specifics.**
4. **Please be aware that Biofire FilmArray does not detect *Stenotrophomonas maltophilia.* If *S. maltophilia* infection is suspected please adjust therapy accordingly in at risk populations.**

© 2018 University College Hospitals NHS Foundation Trust

INHALE

Key

No known allergy to antibiotics
Mild allergy to β–lactams i.e. rash
Severe allergy to β–lactams, i.e. anaphylaxis
Not applicable

*Table 1. To be used when **ONE** Organism is Detected by FilmArray*

First *What organism was found & it the patient allergic to β-lactams*	***Second*** *If NO resistance genes found, this is the advised R_x*	***Third*** *If resistance genes found, this is your advised R_x*			
		**Resistance Marker**			
	None	CTX-M	KPC or OXA-48	IMP, NDM or VIM	*mecA* or *mecC*
No organisms found	Antibiotics should be stopped unless there is clear evidence for probable or proven bacterial infection severe enough to warrant them				
Any virus	Co-amoxiclav +antiviral if appropriate				
	Cefuroxime +antiviral if appropriate				
	Levofloxacin + antiviral if appropriate				

© 2018 University College Hospitals NHS Foundation Trust

	First *What organism was found & it the patient allergic to β-lactams*	***Second*** *If NO resistance genes found, this is the advised* R_x	***Third*** *If resistance genes found, this is your advised* R_x			
		None	CTX-M	KPC or OXA-48	IMP, NDM or VIM	*mecA* or *mecC*
	Any virus + 1 or more bacteria	Treat as indicated for bacterial infection and add antiviral treatment where appropriate				
Organism	*A. baumannii*	Meropenem[1]				
		Meropenem[1]				
		Colistin alone or in combination[2] if clinically appropriate				
	E. aerogenes, E. cloacae, E. coli, K. pneumoniae or K. oxytoca	Temocillin (2g TDS)	Temocillin (2g TDS)	Ceftazidime-avibactam[3]	Colistin alone or in combination[2] if clinically appropriate	
		Ceftriaxone (*Klebsiella* spp. & *E. coli*) OR Meropenem for *Enterobacter* spp.	Meropenem	Ceftazidime-avibactam[3]	Colistin alone or in combination[2] if clinically appropriate	
		Levofloxacin or Ciprofloxacin	Colistin alone or in combination[2] if clinically appropriate	Colistin alone or in combination[2] if clinically appropriate	Colistin alone or in combination[2] if clinically appropriate	
	Proteus spp. or *S. marcescens*	Piperacillin-tazobactam for Serratia sp. OR Temocillin 2g TDS for Proteus sp.	Temocillin 2g TDS for Proteus sp. OR Meropenem for Serratia sp.	Ceftazidime-avibactam[3]	Fosfomycin[4]	
		Ceftriaxone	Meropenem	Ceftazidime-avibactam[3]	Fosfomycin[4]	
		Fosfomycin[4]	Fosfomycin[4]	Fosfomycin[4]	Fosfomycin[4]	
	H. influenzae	Co-amoxiclav				
		Cefuroxime				
		Doxycycline OR Levofloxacin or Ciprofloxacin				

© 2018 University College Hospitals NHS Foundation Trust

	M. catarrhalis	Co-amoxiclav				
		Cefuroxime				
		Doxycycline OR Levofloxacin or Ciprofloxacin				
	P. aeruginosa	Ceftazidime (2g TDS)	Meropenem	Ceftazidime-avibactam[3]	Colistin alone or in combination[2] if clinically appropriate	
		Ceftazidime (2g TDS)	Meropenem	Ceftazidime-avibactam[3]	Colistin alone or in combination[2] if clinically appropriate	
		Colistin alone or in combination[2] if clinically appropriate	Colistin alone or in combination[2] if clinically appropriate	Colistin alone or in combination[2] if clinically appropriate	Colistin alone or in combination[2] if clinically appropriate	
	S. aureus	Flucloxacillin[5]				Glycopeptide[6] or Linezolid
		Cefuroxime				Glycopeptide[6] or Linezolid
		Glycopeptide[6] or Linezolid				Glycopeptide[6] or Linezolid
	S. agalactiae, S. pneumoniae or *S. pyogenes*	Amoxicillin				
		Cefuroxime				
		Glycopeptide[6] or Linezolid				
	C. pneumoniae, L. pneumophila, M. pneumoniae	Macrolide[7] OR Levofloxacin or Ciprofloxacin				
		Macrolide[7] OR Levofloxacin or Ciprofloxacin				
		Macrolide[7] OR Levofloxacin or Ciprofloxacin				

Footnotes

1. In units with high rates of carbapenem resistance, or if experiencing outbreak of carbapenem-resistant *A. baumannii* follow same recommendations as for treatment in case of allergy
2. Colistin can be combined with an appropriate second antimicrobial such as rifampicin or tigecycline. The choice is left open according to local preference.
3. Please discuss with microbiologist before prescribing
4. Consider adding colistin as metallo β-lactamase likely to be present in undetected host organism
5. If clinical picture suggests PVL-positive *S. aureus* consider ordering PVL test and switching to linezolid
6. Vancomycin or teicoplanin
7. Clarithromycin or azithromycin

© 2018 University College Hospitals NHS Foundation Trust

INHALE

Key

No known allergy to antibiotics

Mild allergy to β–lactams i.e. rash

Severe allergy to β–lactams, i.e. anaphylaxis

Not applicable

Table 2. Recommended treatment for combination of* TWO *or more organisms are detected by FilmArray

PLEASE READ THIS TABLE FROM LEFT TO RIGHT; Coloured boxes refer to allergy status as in Table 1.

Key: *+ organism present, - organism absent,* ± either present or absent

**First, What combination of bacteria have been found?**						***Second:***	***Third: if resistance genes found***			
A. baumannii	*Enterobacterales: E. aerogenes, E. cloacae, E. coli, K. pneumoniae ,K. oxytoca, Proteus* sp., *S. marcescens*	*P. aeruginosa*	*H. influenzae/M. catarrhalis*	*S. aureus*	*S. agalactiae, S. pneumoniae* or *S. pyogenes*	**Therapy if no resistance genes**	*mecA/C* found	CTX-M found	*C. pneumoniae, L. pneumophila* OR *M. pneumoniae*	Carbapen-emase found
Does the mixture include *Acinetobacter*? If YES ; stay with this block; if NO, go to next block										
+	Any one or more second organism found					Meropenem[8]	Add Glycopeptide[10] OR Linezolid	-	Add Macrolide[11] OR Levofloxacin or Ciprofloxacin	Discuss with Micro-biology
+	± Any one or more second organism found					Meropenem[8]	Add Glycopeptide[10] OR Linezolid	-	Add Macrolide[11] OR Levofloxacin or Ciprofloxacin	Discuss with Micro-biology
+	Add Levofloxacin or Ciprofloxacin [9]	Add Levofloxacin or Ciprofloxacin [9]	Add Levofloxacin or Ciprofloxacin [9]	Add Glycopeptide[10] OR Linezolid	Add Glycopeptide[10] OR Linezolid	Colistin Combination	Add Glycopeptide[10] OR Linezolid	Discuss with Micro-biology	Add Macrolide[11] OR Levofloxacin or Ciprofloxacin	Discuss with Micro-biology

© 2018 University College Hospitals NHS Foundation Trust

INHALE

**First,** **What combination of bacteria have been found?**						**Second:**	**Third:** *if resistance genes found*			
A. baumannii	*E. aerogenes, E. cloacae, E. coli, K. pneumoniae, K. oxytoca, Proteus* sp., *S. marcescens*	*P. aeruginosa*	*H. influenzae/M. catarrhalis*	*S. aureus*	*S. agalactiae, S. pneumoniae* or *S. pyogenes*	**Therapy if no resistance genes**	*mecA/C* found	CTX-M found	*C. pneumoniae, L. pneumophila* OR *M. pneumoniae*	Carbapenemase found
If NO *Acinetobacter*: but ≥1 *Pseudomonas*/Enterobacteriales found start here										
-	+ (at least one)		±	±	±	Piperacillin/Tazobactam	Add Glycopeptide[10] OR Linezolid	Escalate to Meropenem	Add Macrolide[11] OR Levofloxacin or Ciprofloxacin	Discuss with Micro-biology
-	+ (at least one)		±	±	±	Meropenem	Add Glycopeptide[10] OR Linezolid	-	Add Macrolide[11] OR Levofloxacin or Ciprofloxacin	Discuss with Micro-biology
-	Add Levofloxacin or Ciprofloxacin [9]	Add Levofloxacin or Ciprofloxacin[9]	Add Levofloxacin or Ciprofloxacin[9]	Add Glycopeptide[10] OR Linezolid	Add Glycopeptide[10] OR Linezolid	Colistin Combination as indicated	Add Glycopeptide[10] OR Linezolid	Discuss with Micro-biology	Add Macrolide[11] OR Levofloxacin or Ciprofloxacin	Discuss with Micro-biology
If NO *Acinetobacter* NO *Pseudomonas* & NO Enterobacteriaceae start here										
None of these			Any 2 or more of these			Co-amoxiclav	Add Glycopeptide[10] OR Linezolid		Add Macrolide[11] OR Levofloxacin or Ciprofloxacin	
None of these			Any 2 or more of these			Levofloxacin	Add Glycopeptide[10] OR Linezolid		Add Macrolide[11] OR Levofloxacin or Ciprofloxacin	
None of these			Any 2 or more of these			Levofloxacin	Add Glycopeptide[10] OR Linezolid		Add Macrolide[11] OR Levofloxacin or Ciprofloxacin	

Footnotes

8. Add colistin in in areas of high carbapenem-resistance among *A. baumanii.*
9. Consider adding tigecycline of fosfomycin if fluoroquinolone resistance locally prevalent
10. Vancomycin or teicoplanin
11. Clarithromycin or azithromycin

© 2018 University College Hospitals NHS Foundation Trust
