# Supplementary material for: INHALE: the impact of using FilmArray Pneumonia Panel molecular diagnostics for hospital-acquired and ventilator-associated pneumonia on antimicrobial stewardship and patient outcomes in UK Critical Care—study protocol for a multicentre randomised controlled trial
Source: Trials. 2021 Oct 7;22:680. doi: 10.1186/s13063-021-05618-6 (PMC8496625; doi:10.1186/s13063-021-05618-6)
Supplement: Supplementary file 3 — Additional file 3: Table S1. Timeline for Trial Actions. *Later on day 1 and as soon as possible after decision to test for pneumonia. Further specimens from the same participant may be tested on the machine, within the 21-day trial period, only if clinically indicated and a sample has also been sent routinely to the microbiology laboratory. 1Every day until 14 days after randomisation or until clinical cure of pneumonia, whichever is first. Assessments only required on these days if in ICU/CCU and not cured of pneumonia. 2Which assessment is used depends on whether participant is a child or an adult. Clinical teams record these routinely and will know which should be used. 3Data collected may pre- and post-date the trial period, but will be collected from clinical records by hospital staff. 4Window for “day 21” phone call to occur is from days 20-24 post randomisation, discharged participants only. 5Record if relevant until 21 days after randomisation (even if pneumonia is cured sooner). [file 13063_2021_5618_MOESM3_ESM.docx]

Table 1: Timeline for Trial Actions

| **Timeline for trial actions** | **Screening and Baseline (may occur on D1)** | **Day 1** | **Day 2** | **Day 3** | **Day 4** | **Daily assessment** | **Day 14** | **Daily assessment^5^** | **Day 21**  **(-1/+3 days)** | **Day 28** |
| --- | --- | --- | --- | --- | --- | --- | --- | --- | --- | --- |
| Clinician agrees participant can be considered for the trial | X |  |  |  |  |  |  |  |  |  |
| Check eligibility | X |  |  |  |  |  |  |  |  |  |
| Demographics added to database | X |  |  |  |  |  |  |  |  |  |
| Routine specimen taken for HAP/VAP, split to provide 2 samples |  | X |  |  |  |  |  |  |  |  |
| Randomisation |  | X |  |  |  |  |  |  |  |  |
| Sample 1 sent for routine microbiology testing |  | X |  |  |  |  |  |  |  |  |
| Sample 2 (intervention arm) tested on FilmArray machine |  | X |  |  |  |  |  |  |  |  |
| Sample 2 (control arm) stored in -20^o^C (or lower) freezer |  | X |  |  |  |  |  |  |  |  |
| Review and act on machine result when available (intervention only) |  | X* |  |  |  |  |  |  |  |  |
| Review and act on microbiology result (both groups) when available |  |  |  |  |  |  |  |  |  |  |
| Medical history including current antibiotic use^3^ |  | X |  |  |  |  |  |  |  |  |
| Apache2/PIM3^2^ (as recorded on admission for current admission) | X |  |  |  |  |  |  |  |  |  |
| SOFA/Paediatric SOFA/PELOD-2^2^ and infection markers on day 1 |  | X | X | X^1^ | X^1^ | X^1^ | X^1^ |  | X |  |
| Adverse events (including c.diff) septic shock (if relevant)^5^ |  | X | X | X | X | X | X | X | X |  |
| Mortality to be recorded (if relevant) |  | X | X | X | X | X | X | X | X | X |
| Ventilation details (if relevant)^5^ |  | X | X | X | X | X | X | X | X |  |
| Antimicrobial prescriptions given^5^ |  | X | X | X | X | X | X | X | X |  |
| Pneumonia status^5^ |  | X | X | X | X | X | X | X | X |  |
| Is Routine chest x-ray and/or CT scan available and does it show pneumonia? | X |  |  |  |  |  | X |  | X |  |
| EuroQoL EQ-5D-5L collected (if relevant) |  |  |  |  |  |  |  |  | X |  |
| Hospital resource use^3^ collected | X |  |  |  |  |  |  |  | X |  |
| Follow up phone call (if relevant) |  |  |  |  |  |  |  |  | X^4^ |  |

*Later on day 1 and as soon as possible after decision to test for pneumonia. Further specimens from the same participant may be tested on the machine, within the 21-day trial period, only if clinically indicated and a sample has also been sent routinely to the microbiology laboratory

^1^Every day until 14 days after randomisation or until clinical cure of pneumonia, whichever is first. Assessments only required on these days if in ICU/CCU and not cured of pneumonia

^2^Which assessment is used depends on whether participant is a child or an adult. Clinical teams record these routinely and will know which should be used

^3^Data collected may pre- and post-date the trial period, but will be collected from clinical records by hospital staff

^4^Window for “day 21” phone call to occur is from days 20-24 post randomisation, discharged participants only

^5^Record if relevant until 21 days after randomisation (even if pneumonia is cured sooner)
