# Supplementary material for: INHALE: the impact of using FilmArray Pneumonia Panel molecular diagnostics for hospital-acquired and ventilator-associated pneumonia on antimicrobial stewardship and patient outcomes in UK Critical Care—study protocol for a multicentre randomised controlled trial
Source: Trials. 2021 Oct 7;22:680. doi: 10.1186/s13063-021-05618-6 (PMC8496625; doi:10.1186/s13063-021-05618-6)
Supplement: Supplementary file 4 — Additional file 4:. INHALE Micro Committee Terms of Reference v2.0 27JUN2021. [file 13063_2021_5618_MOESM4_ESM.pdf]

INHALE MICROBIOLOGY COMMITTEE

Author	Position	Signature	Date
Dr Virve Enne	INHALE Programme Manager (Committee Chair)		
**Committee Members**	**Position**	**Signature**	**Date**
Prof David Livermore	INHALE WP3 Study Team voting member		
Dr Benny Cherian	Independent voting member		
Dr Matthew Dryden	Independent voting member		
Dr Peter Riley	Independent voting member		
Dr Vanya Gant	Chief Investigator (Presenter)		

All signatories agreed to sign the document on 28th July 2021

TERMS OF REFERENCE v2.0 27JUL2021

1. The Committee will comprise 4 voting members: 1 from the INHALE team (Prof. David Livermore) and 3 independents (Drs Matthew Dryden, Benny Cherian and Peter Riley). It will be chaired by Dr Vicky Enne (non-voting). Additional non-voting attendees are Dr Vanya Gant who presents clinical cases and Ms. Charlotte Russell taking notes.

2. The Committee meets *c.* monthly via teleconference. Each meeting lasts 2h and handles on average 15-20 patients per meeting. Longer face-to-face meetings will be held towards the end of the study to clear backlog. Each meeting will handle a balance of patients from both study arms.

3. The Committee will be blinded to the study arm of each patient when making categorisation decisions. In practice this will be achieved through production of uniform data reports for both arms by Norwich Clinical Trials Unit (NCTU). Actual study numbers will be obscured to avoid identification of the study arm.

4. Dr Gant will present each case based on the report produced by NCTU. His outline will detail:

    a. FilmArray and routine microbiology results. ALL results by both methods will be considered for each patient, irrespective of their trial arm. FilmArray results for control arm patients will be generated centrally by the study team and treating clinicians would not have been aware of them.

    b. The patient's clinical condition and co-morbidities at diagnosis of HAP/VAP.

    c. Details of antimicrobial prescribed for the pneumonia.

    d. Details of confounders including other concurrent infections and antibiotics prescribed for these.

    e. Details of eventual patient outcomes will not be included to avoid influencing decisions.

5. The Committee will decide collectively whether antimicrobial prescribing for the HAP or VAP was i) microbiologically active and ii) proportionate at 24h and 72h post-randomisation. i.e. 4 primary decisions will be made per patient.

6. In the case of disagreement among Committee members, the decision will be referred for external adjudication by Dr. Ruan Simpson, who does not participate in meetings. Dr. Simpson will also independently adjudicate 10% of decisions as a quality control measure, to ensure the robustness of decision making by the Committee.

7. In addition, the Committee will categorise all relevant antimicrobials (or their combinations) as ‘broad-spectrum’ or ‘narrow-spectrum’. A small number of older broad-spectrum antibiotics that are now heavily compromised by resistance (e.g. co-trimoxazole, tetracycline and chloramphenicol) are assigned a separate category as “old”. The resulting list will be used to answer the secondary question of whether the patient was on narrow-spectrum antimicrobials at 24h and 72h. Patients not on antibiotics at these time points are noted.

8. In addition to the primary and secondary outcomes listed in points 5 and 7 above, the Committee also makes a note of a number of additional factors that influence their decisions. These are

    a. Patients with no positive microbiology or FimArray results (completely negative result).
    b. For patients with no positive microbiology the Committee considers whether the clinical condition is severe enough to warrant continuation of antimicrobial therapy.
    c. Patients in whom the only pathogen found is a virus.
    d. Patients in whom no antimicrobial therapy is justified because they are no longer thought to be infected at 24h and/or 72h.
    e. Patients for whom no decision can be reached because data are unlikely to be obtainable, owing e.g. to death or withdrawal. The reason is noted.
    f. Patients in whom either the FilmArray or routine microbiology result led to a concerning antimicrobial therapy being administered are noted and reported to the Data Monitoring Committee. These particularly include any case where the algorithm has or may have prompted use of an agent to which the pathogen ultimately found proves to be resistant.
    g. Patients whose case reports suggests they did not meet eligibility criteria of the study.

9. In achieving their decisions regarding activity and proportionality, the Committee will be guided the following general principles:

    a. Antibiotics will be considered inactive when they are found inactive *in-vitro* against the pathogen isolated, where the pathogen (if found by FilmArray. only) has a gene that ordinarily causes resistance or where an inherently resistant pathogen is found.

    b. Antibiotics will be considered active where they are found active *in-vitro* against the pathogen isolated. If a pathogen is detected by FilmArray only, and no susceptibility results are available, the Committee will make assumptions regarding likely activity based on local and national surveillance data.

    c. Proportionality will be judged on the basis whether the antibiotic has an unnecessarily broad spectrum for the pathogen(s) detected (e.g. meropenem *vs. H. influenzae*).

d. Any antimicrobial that is judged to be inactive against the pathogens found will automatically also be disproportionate.

e. The antimicrobial advocated on the master prescribing algorithm or its accepted local variants will be counted as active and proportionate against the pathogen specified on the algorithm unless the particular isolate proves to be resistant when grown [NB this means that the Committee must accept the algorithm as reasonable].

f. In cases where multiple organisms are found at different concentrations by FilmArray testing, the Committee may take the view that organism(s) present at low concentrations may be colonists that do not warrant treatment. For the time being, such decisions will be made on a case-by-case basis.

g. For patients with completely negative microbiology results, the decision on whether prescribing is clinically appropriate and proportionate will be made in the context of the patient's clinical parameters. Such patients will be identified separately in the database, allowing them to be distinguished during analyses. For example, it would be considered appropriate and proportionate for a severely ill patient to receive broad spectrum treatment, but it would be inappropriate and disproportionate in a patient with mild infection.

h. Infections at other body sites, and antibiotics given to treat these, will be considered on a case-by-case basis.

10. The Committee's collective decisions for each recruited patient will be recorded manually during the meeting by a non-expert member of NCTU or the broader study team. They will be matched with study numbers and entered on the REDCap database at a later date.
